# Dose–response effects of exercise and caloric restriction on visceral adiposity in overweight and obese adults: a systematic review and meta-analysis of randomised controlled trials

**DOI:** 10.1136/bjsports-2022-106304

**Published:** 2023-01-20

**Authors:** Francesco Recchia, Chit K. Leung, Angus P. Yu, Welton Leung, Danny J. Yu, Daniel Y. Fong, David Montero, Chi-Ho Lee, Stephen H.S. Wong, Parco M. Siu

**Affiliations:** 1 Division of Kinesiology, School of Public Health, Li Ka Shing Faculty of Medicine, The University of Hong Kong, Hong Kong, Hong Kong; 2 Department of Psychiatry, The Chinese University of Hong Kong, Hong Kong, Hong Kong; 3 School of Nursing, Li Ka Shing Faculty of Medicine, The University of Hong Kong, Hong Kong, Hong Kong; 4 Department of Medicine, The University of Hong Kong, Hong Kong, Hong Kong; 5 Department of Sports Science and Physical Education, The Chinese University of Hong Kong, Hong Kong, Hong Kong

**Keywords:** Exercise

## Abstract

**Objective:**

To determine and compare the dose–response effects of exercise and caloric restriction on visceral adipose tissue in overweight and obese adults, while controlling for the weekly energy deficit induced by the interventions.

**Methods:**

PubMed, Embase, CINAHL and Web of Science were searched for randomised controlled trials comparing exercise or caloric restriction against eucaloric controls in overweight or obese adults. The primary outcome was the change in visceral fat measured by CT or MRI. Meta-analyses and meta-regressions were performed to determine the overall effect size (ES) and the dose–dependent relationship of exercise and caloric restriction on visceral fat. Heterogeneity, risk of bias and the certainty of evidence were also assessed.

**Results:**

Forty randomised controlled trials involving 2190 participants were included. Overall, exercise (ES −0.28 (−0.37 to −0.19); p<0.001; I^2^=25%) and caloric restriction (ES −0.53 (−0.71 to −0.35); p<0.001; I^2^=33%) reduced visceral fat compared with the controls. Exercise demonstrated a dose–response effect of −0.15 ((−0.23 to −0.07); p<0.001) per 1000 calories deficit per week, whereas the effect of caloric restriction was not dose-dependent (ES 0.03 (−0.12 to 0.18); p=0.64). Most of the studies showed a moderate risk of bias.

**Conclusions:**

These findings support the dose–dependent effects of exercise to reduce visceral fat in overweight and obese adults. Caloric restriction did not demonstrate a dose–response relationship, although this may be attributed to the smaller number of studies available for analysis, compared with exercise studies.

**PROSPERO registration number:**

CRD42020210096.

**WHAT IS ALREADY KNOWN ON THIS TOPIC**

- Obesity management guidelines recommend the use of exercise and caloric restriction for weight loss in obese individuals. However, the comparative effectiveness of exercise and caloric restriction interventions on visceral fat changes has not been established.

**WHAT THIS STUDY ADDS**

- Both interventions can effectively reduce visceral fat of overweight and obese individuals. However, only exercise showed a dose–dependent relationship between energy expenditure and visceral fat.

**HOW THIS STUDY MIGHT AFFECT RESEARCH, PRACTICE OR POLICY**

- Obesity management guidelines should consider the dose-dependent effects of exercise as an effective lifestyle interventional strategy to reduce visceral fat in overweight and obese adults. Further research is needed to elucidate the effects of caloric restriction on visceral fat.

## Background

Obesity as a worldwide pandemic continues to demonstrate a growing prevalence. According to the WHO, 39% of adults worldwide were overweight and 13% were obese in 2016.^1^ It is well documented that obesity is a key contributor to cardiovascular disease, type 2 diabetes mellitus, metabolic syndrome, cancer and other chronic diseases.^2–6^ Over the past decades, internationally recognised obesity management guidelines have been developed to promote lifestyle interventional strategies incorporating regular exercise and caloric restriction.^7–10^ These recommendations are primarily designed to reduce body weight, as an elevated body mass index (BMI) is clinically used to characterise overweight and obesity according to the WHO cut-offs.^11^

Although BMI satisfactorily correlates with body fat percentage when adjusted for sex, age and ethnicity,^12^ it has been shown that visceral fat presents a far greater cardiometabolic risk than subcutaneous fat,^13^ and thus BMI is not entirely indicative of the risk for cardiometabolic diseases, as it cannot reflect individual variability in fat deposition.^14^ A recent joint position statement from the International Atherosclerosis Society and the International Chair on Cardiometabolic Risk Working Group on Visceral Obesity supported the notion that visceral fat is an independent risk factor for cardiovascular and metabolic morbidity and mortality, whereas BMI fails to determine cardiometabolic risk.^15^ This suggests that visceral fat might be a more important indicator of the efficacy of obesity management strategies.

A previous meta-analysis compared the effects of exercise and caloric restriction on reducing visceral fat, but made only head-to-head comparisons based on a small number of studies with both exercise and dietary intervention arms (n=8).^16^ The meta-analysis indicated a trend towards a greater reduction in visceral fat following exercise, but this conclusion was based on within-group pre–post changes rather than comparing to non-exercising controls.^16^ The independent effects of exercise versus caloric restriction on visceral fat, when compared with eucaloric conditions and while controlling for weekly energy deficit, remain unknown. Earlier evidence suggests that both exercise and caloric restriction produce dose–response effects.^17 18^ However, a previous randomised controlled trial indicated a preferential reduction in visceral fat with exercise over caloric restriction.^19^ Although both are established lifestyle strategies for the prevention and management of obesity, the physiological and metabolic adaptations to exercise and caloric restriction are fundamentally different.^20 21^ These differences might also reflect distinct responses in reducing visceral fat. Assessing the dose–response effects of exercise and caloric restriction is therefore crucial to provide insights on the potential cumulative effects of these interventions for maximising visceral fat loss in overweight and obese people. This study aimed to determine and compare the dose–response effects of exercise and caloric restriction on visceral fat in overweight and obese adults, while controlling for weekly caloric deficit induced by either an increase in energy expenditure via exercise or a decrease in energy intake via caloric restriction.

## Methods

This review conformed to the Preferred Reporting Items for Systematic Reviews and Meta-Analyses and was registered in PROSPERO (CRD42020210096).

### Data sources and eligibility criteria

PubMed, Embase, CINAHL and Web of Science were searched for relevant articles written in any language from inception to the search date. Details regarding the search terms used are available in online supplemental appendix S1. The reference lists of relevant meta-analyses and articles of interest were also screened. One independent reviewer performed the search on January 2021 and a second independent reviewer repeated the search on January 2022. Any disagreements between the first and the second reviewers were resolved by consensus.

10.1136/bjsports-2022-106304.supp1 Supplementary data

We included randomised controlled trials comparing exercise or caloric restriction with eucaloric controls in overweight or obese adults (≥18 years old). Overweight and obesity were defined using either the WHO cut-off scores for BMI^11^ or the waist circumference standards set by the International Diabetes Federation.^6^

### Outcomes

The primary outcome of this study was the change in visceral fat from baseline, quantified by CT or MRI, which are both considered to be gold-standard methods.^22^ Studies that assessed visceral fat by other methods were excluded. The secondary outcome was the change in waist circumference. No specific criteria were set for the measurement protocol of waist circumference.

### Data extraction and quality assessment

To calculate effect sizes (ESs), two independent reviewers extracted sample sizes, changes in visceral fat and/or waist circumference from baseline and SD, for every study. Any disagreements were resolved by consensus. When SDs were not reported, we used previously validated methods to calculate them.^23 24^ If other information was missing, an attempt was made to contact the study investigators to obtain the necessary data. If the study authors were unresponsive or unreachable, the study was excluded.

Data related to the study (first author, date, country), the participants (mean age, sex, comorbidities) and the intervention (type of intervention, frequency, intensity, time and volume of the exercise intervention, diet prescription, intervention duration and the method used to quantify visceral fat) were also extracted.

Risk of bias was assessed using the Cochrane’s Risk of Bias 2 tool.^25^ Bias was assessed in the following domains: (1) bias arising from the randomisation process, (2) bias due to deviations from the intended interventions, (3) bias due to missing outcome data, (4) bias in the measurement of the outcomes and (5) bias in the selection of the reported result. Two reviewers independently performed the risk of bias assessment. Disagreements were resolved by discussion. The Grading of Recommendations, Assessment, Development and Evaluations (GRADE) approach was adopted to assess the certainty in the body of evidence on exercise and caloric restriction interventions for visceral fat reduction.^26^

### Data synthesis and analysis

Statistical analyses were conducted using the metafor package in the statistical software R (V.4.2.0).^27^ Statistical significance was set at p<0.05.

ESs were synthesised as d_pcc2_ as proposed by Morris;^28^ namely, the mean change from baseline in the control group was subtracted from the mean change in the intervention group, and the difference was divided by the baseline pooled SD and multiplied by a bias adjustment for small sample size. The formula below was used to calculate the bias adjustment:

$$\sqrt{\frac{2}{df}}\left(\frac{\Gamma \left[df/2\right]}{\Gamma \left[\left(df-1\right)/2\right]}\right)$$

where Γ is the gamma function.^29^ This method was reported to produce better results in terms of bias, precision and robustness to heterogeneity of variance.^28^ ESs and CIs were aggregated using the inverse variance model and the Sidik-Jonkman variance estimator with the Hartung-Knapp modification.^30–32^ A negative ES indicated a beneficial effect of the main intervention over the comparison group.

Heterogeneity was assessed using I^2^ and interpreted as follows: 0%–40%, might not be important; 30%–60%, may represent moderate heterogeneity; 50%–90%, may represent substantial heterogeneity; and 75%–100%, considerable heterogeneity.^32^

Meta-regression was performed to explore the dose–response relationship of exercise and caloric restriction on reducing visceral fat, and to determine the potential superiority of one intervention over the other while controlling for weekly energy deficit. To determine the dose–response relationship of the two interventions, weekly energy deficit was used as an effect modifier. When energy deficits were not provided, they were computed from the available rates of metabolic equivalent or measures of oxygen uptake.^33 34^ If energy deficits could not be computed, the study was included in the meta-analysis but excluded from the meta-regression analyses. The comparison between exercise and caloric restriction was assessed by including an interaction term in the meta-regression model.

Secondary meta-regressions were performed to explore the potential influence of participant, intervention and study characteristics on the overall effects. Exercise frequency, intensity, session duration, intervention duration, supervision, method to quantify visceral fat, study continent, comorbidities, age, baseline BMI and sex ratio were selected as effect modifiers using univariate meta-regression and subgroup analyses. Exercise frequency was treated as both a continuous and categorical variable (>3.5 or ≤3.5 days/week). Exercise intensity was categorised according to the American College of Sports Medicine’s guidelines.^33^ Details regarding the categorisation of exercise intensity are available in online supplemental table S1A–S1B).

Influential analyses were performed to identify possible outliers.^27^ Analyses for the primary outcome were repeated after removing influential observations and after removing studies where weekly energy deficits were not provided and had to be calculated and studies with high risk of bias.

### Equity, diversity and inclusion statement

The author group consists of junior, mid-career and senior researchers from different countries and disciplines. Our study population included both male and female adults from different socioeconomic and cultural backgrounds; thus, our findings may be generalisable to a wide range of individuals.

## Results

### Search results

The electronic database search identified 7816 unique records. After assessment for eligibility, 54 records comprising 40 studies were included (online supplemental figure S1). Due to missing outcome data, four studies were excluded from the meta-analysis, but were still included in the systematic review. Of the 36 studies included for meta-analysis, 5 studies were added twice because they had both an exercise and a caloric restriction arm. Overall, 26 studies (k=46) were analysed for exercise and 15 studies (k=16) were analysed for caloric restriction.

**Figure 1:**
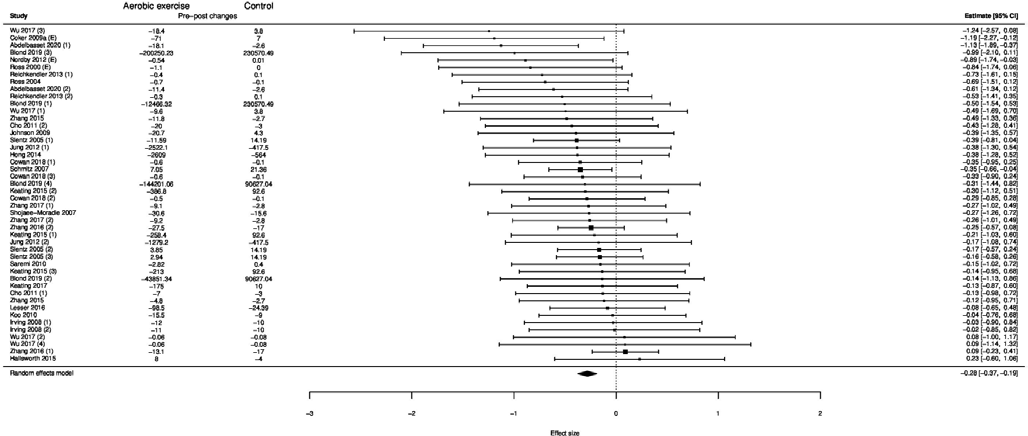
Forest plot of the effect of exercise on visceral fat.

### Study characteristics

A summary of the characteristics of the included studies is presented in table 1A,1B. Further information regarding the study and intervention characteristics is provided in online supplemental table S2A–S2B. The majority of studies were conducted in the USA (n=15), Asia (n=11) and Europe (n=9). Overall, 2190 participants were assigned to exercise (n=983), caloric restriction (n=394) and control (n=813). Eight studies included individuals with comorbidities, such as type 2 diabetes, metabolic syndrome, dyslipidaemia and non-alcoholic fatty liver disease. The exercise interventions ranged from 4 weeks to 2 years, whereas caloric restriction interventions ranged from 12 weeks to 1 year. Half of the studies measured visceral fat using MRI and the other half used CT.

**Table 1:** (A) characteristics of exercise studies and (B) characteristics of caloric restriction studies

Study	Comorbidity	Age* (years)	BMI* (kg/m^2^)	WC* (cm)	Duration (wk)	Groups†	N (M/F)	Measure
A								
Abdelbasset *et al* 63–65 2019, 2020a, b * †	NAFLD, T2DM	40–60	≥30	NR	8	HIIT (1) MICT (2) CON	16 (10/6) 15 (8/7) 16 (9/7)	MRI‡
Blond *et al* 41 2019	None	20–45	25–35	NR	24	MOD (1) VIG (2) CON	23 (12/11)§ 17 (7/10)§ 12 (6/6)§	MRI
Cho *et al* 42 2011	None	34–60	≥25	NR	12	HI (1) LI (2) CON	12 (0/12) 13 (0/13) 10 (0/10)	CT
Coker *et al* 35 2009*	None	50–80	26 to <40	NR	12	AE CON	9 (3/6) 8 (3/5)	CT
Coker *et al* 35 2009† ¶	None	65–90	26 to <37	NR	12	HI (1) MI (2) CON	6 (3/3) 6 (3/3) 6 (3/3)	CT
Cowan *et al* 40 2018	None	35–65	NR	M>102 F>88	24	LILV (1) LIHV (2) HIHV (3) CON	24 (14/10) 31 (20/11) 40 (19/11) 20 (10/10)	MRI
Davidson *et al* 66 2009¶	None	60–80	27–34.9‡	M≥102 F≥88	24	AE CON	37 (17/20) 28 (11/17)	MRI
Hallsworth *et al* 67 2015	NAFLD	30–70‡	25–35‡	NR	12	HIIT CON	12 (NR) 11 (NR)	MRI
Hong *et al* 68 2014	None	30–40	>25	NR	12	AE CON	10 (0/10) 10 (0/10)	CT
Irving *et al* 69 70 2008, 2009	MetS	MA	NR	IDF	16	HI (1) LI (2) CON	11 (3/8) 13 (3/10) 10 (4/6)	CT
Johnson *et al* 29 2009	None	>18§	≥30	NR	4	AE CON	12 (NR) 7 (NR)	MRI
Jung *et al* 71 2012	T2DM	45–65	>23	NR	12	MOD (1) VIG (2) CON	8 (0/8) 8 (0/8) 12 (0/12)	CT
Keating *et al* 72 2015	Pre-diabetes‡	29–59	>25	NR	8	HILV (1) LIHV (2) LILV (3) CON	12 (6/6) 12 (5/7) 12 (3/9) 12 (3/9)	MRI
Keating *et al* 73 2017	Pre-diabetes‡	29–59	≥25	NR	8	RT CON	15 (2/13) 14 (2/12)	MRI
Koo *et al* 39 2010	T2DM	> 18	>23	NR	12	AE CON	13 (0/13) 18 (0/18)	CT
Lee *et al* 74 2012¶	None	30–50	>25	>80	14	HI (1) LI (2) CON	7 (0/7) 8 (0/8) 7 (0/7)	CT
Lesser *et al* 75 2016	None	PM	NR	≥80	12	AE CON	23 (0/23) 26 (0/26)	CT
Nordby *et al* 38 2012 Bladbjerg *et al* 76 2017	None	20–40	25–30	NR	12	AE CON	12 (12/0) 12 (12/0)	MRI
Pugh *et al* 77 2014¶ Cuthbertson *et al* 78 2016¶	NAFLD	20–65‡	27–35‡	NR	16	AE CON	30 (23/7) 20 (16/4)	MRI
Reichkendler *et al* 79 2013	None	20–40	25–30	NR	11	HV (1) MV (2) CON	14 (14/0) 13 (13/0) 9 (9/0)	MRI
Ross *et al* 36 2000 Thong *et al* 80 2000	None	>18§	>27	>100	12	AE CON	16 (16/0) 8 (8/0)	MRI
Ross *et al* 37 2004	None	>18	>27	>88	14	AE CON	17 (0/17) 10 (0/10)	MRI
Saremi *et al* 81 2010	None	MA	≥25	NR	12	AE CON	11 (11/0) 10 (10/0)	CT
Schmitz *et al* 82 2007	None	25–44	25–35	NR	96	RT CON	82 (0/82) 82 (0/82)	CT
Shojaee-Moradie *et al* 83 2007	None	>18§	25–30	NR	6	AE CON	10 (10/0) 7 (7/0)	CT
Slentz *et al* 84 2005	Dyslipidaemia	40–65	25–35	NR	24	HIHV (1) HILV (2) MILV (3) CON	42 (23/19) 46 (23/23) 40 (22/18) 47 (23/24)	CT
Wu *et al* 85 2017	None	30–50	≥30	NR	12	HI (1) LI (2) CON	14 (0/14) 11 (0/11) 12 (0/12)	CT
Zhang *et al* 86 2015	None	NR	≥25	NR	12	HIIT (1) MICT (2) CON	12 (0/12) 12 (0/12) 11 (0/11)	CT
Zhang *et al* 87 2016	NAFLD	40–65	NR	M≥90 F≥85	24	MOD (1) VIG (2) CON	73 (22/51) 73 (21/52) 74 (28/46)	CT
Zhang *et al* 88 2017	None	18–22	≥25	NR	12	HIIT (1) MICT (2) CON	15 (0/15) 15 (0/15) 13 (0/13)	CT
B								
Bouchonville *et al* 89 2014 Napoli *et al* 90 2014	Mild-to-moderate frailty	≥65	≥30	NR	48	CR CON	26 (9/17) 27 (9/18)	MRI
Brennan *et al* 91 2021	None	60–80	≥30	NR	24	CR CON	21 (7/14) 20 (7/13)	MRI
Coker *et al* 35 2009*	None	50–80	26 to <40	NR	12	CR CON	9 (3/6) 8 (3/5)	CT
Ibáñez *et al* 92 2010 Idoate *et al* 93 2011 García-Unciti *et al* 94 2012	None	40–60	30–40	NR	16	WL CON	12 (0/12) 9 (0/9)	MRI
Kang *et al* 95 2018	None	20– 65‡	25 to <30	NR	12	LCD CON	47 (13/34) 50 (14/36)	CT
Koo *et al* 39 2010	T2DM	> 18	> 23	NR	12	CR CON	19 (0/19) 18 (0/18)	CT
Larson-Meyer *et al* 96 97 2006, 2010 Redman *et al* 98 99 2007, 2010	None	25–50 M 25–45 F	25–30	NR	24	CR CON	12 (6/6) 11 (5/6)	CT
Lee *et al* 100 2018	None	20–60	25 to <30	NR	12	WL WM	37 (15/22) 38 (11/27)	CT
Ng *et al* 101 102 2007, 2009 Chan *et al* 103 2008	MetS	> 18§	NR	IDF	14	WL WM	20 (20/0) 15 (15/0)	MRI
Nordby *et al* 38 2012 Bladbjerg *et al* 76 2017	None	20–40	25–30	NR	12	CR CON	12 (12/0) 12 (12/0)	MRI
Ross *et al* 36 2000 Thong *et al* 80 2000	None	>18§	>27	>100	12	CR CON	14 (14/0) 8 (8/0)	MRI
Ross *et al* 37 2004	None	>18	>27	>88	14	WL CON	15 (0/15) 10 (0/10)	MRI
Schübel *et al* 104 2018	None	35–65	25 to <40	NR	12	CR CON	48 (NR) 49 (NR)	MRI
Schutte *et al* 105 2022	None	40–70	>27	M>102 F>88	12	LNCR (1) HNCR (2) CON	39 (16/23) 34 (15/19) 27 (12/15)	MRI
Trepanowski *et al* 106 2018	None	18–65	25 to <40	NR	24	CR CON	29 (6/23) 25 (4/21)	MRI

*Inclusion criteria unless otherwise specified.

†Study arms being synthesised.

‡Retrieved from clinical trial registration.

§Ascertained from study investigators.

¶Not included in the meta-analysis due to insufficient data.

AE, aerobic exercise; BMI, body mass index; CON, control group; CR, caloric restriction; EX, exercise; HI, high intensity; HIIT, high-intensity interval training; HN, High nutrient; HV, high volume; IDF, International Diabetes Federation; LCD, low-calorie diet; LI, low intensity; LN, Low nutrient; LV, low volume; MA, middle aged; MetS, metabolic syndrome; MetS, metabolic syndrome; MICT, moderate-intensity continuous training; MI/MOD, moderate intensity; MV, moderate volume; NAFLD, non-alcoholic fatty liver disease; NR, not reported; PM, postmenopausal; RT, resistance training; T2DM, type II diabetes mellitus; VIG, vigorous intensity; WC, waist circumference; WL, weight loss; WM, weight maintenance.

### Effect of exercise on visceral fat and waist circumference

**Figure 2:**
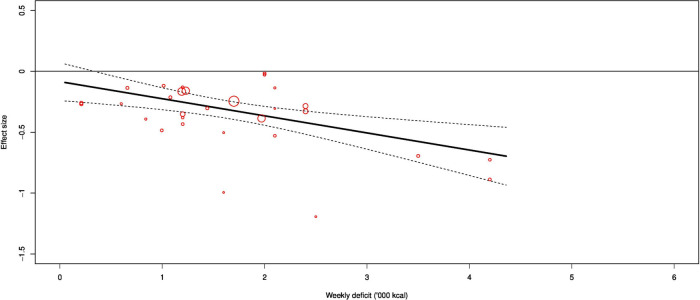
Dose–response effect of exercise on visceral fat.

Exercise significantly reduced visceral fat ((ES) −0.28 (−0.37 to −0.19); p<0.001; I^2^=25%) compared with controls (figure 1). Meta-regression demonstrated a dose–response effect of −0.15 ((−0.23 to −0.07); p<0.001) per 1000 calories deficit per week (figure 2).

Exercise produced an effect of −0.41 ((−0.60 to −0.22); p<0.001; I^2^=43%) on waist circumference (online supplemental figure S2), equivalent to a mean difference of 3.15 cm. Meta-regression showed a dose-response effect of −0.27 ((−0.41 to −0.13); p<0.001) per 1000 calories deficit per week (online supplemental figure S3).

**Figure 3:**
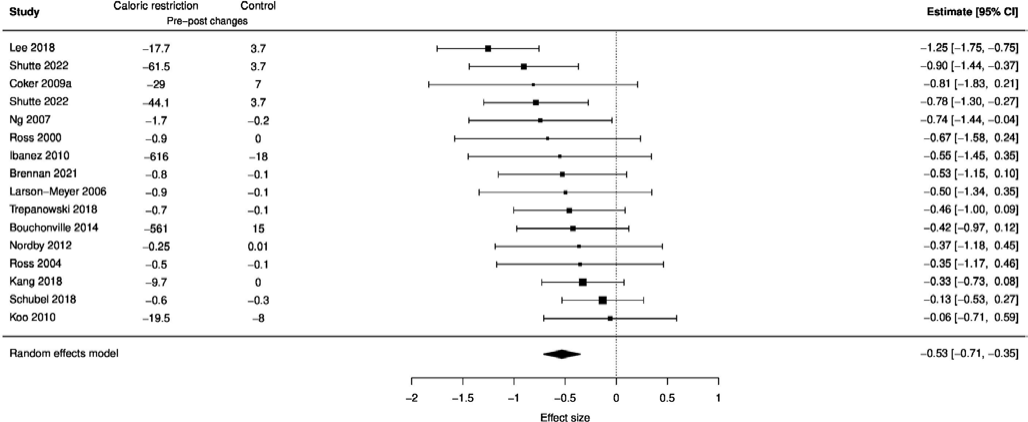
Forest plot of the effect of caloric restriction on visceral fat.

### Effect of caloric restriction on visceral fat and waist circumference

**Figure 4:**
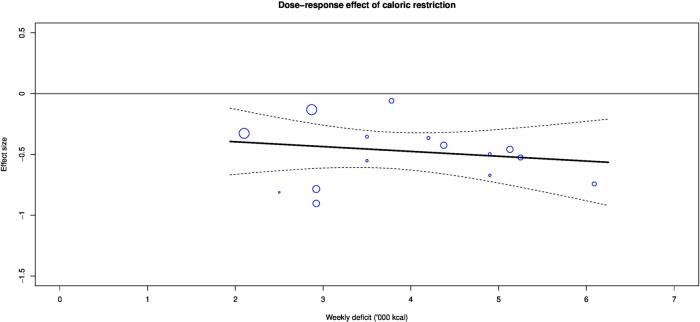
Dose–response effect of caloric restriction on visceral fat.

Caloric restriction significantly reduced visceral fat (ES −0.53 (−0.71 to −0.35); p<0.001; I^2^=33%) compared with controls (figure 3). Meta-regression showed that the effect of caloric restriction was not dose-dependent (ES 0.03 (−0.12 to 0.18); p=0.64) (figure 4).

Caloric restriction produced an effect of −0.59 ((−1.03 to −0.16); p=0.013; I^2^=76%) on waist circumference (online supplemental figure S4), equivalent to a mean difference of 4.67 cm. The effect of caloric restriction on waist circumference was dose-dependent (ES −0.29 (−0.58 to −0.00); p=0.048) (online supplemental figure S5).

### Effect of exercise versus caloric restriction on visceral fat

Exercise and caloric restriction were compared via meta-regression using weekly energy deficit and the type of treatment as effect modifiers. The results showed that exercise had a superior dose–response effect on reducing visceral fat compared with caloric restriction (ES −0.18 (−0.33 to −0.04); p=0.012).

### Risk of bias and GRADE

For the exercise studies, risk of bias was rated low in 6 studies, moderate in 14 studies and high in 5 studies (online supplemental figure S6A). For the caloric restriction studies, risk of bias was rated low in 3 studies, moderate in 10 studies and high in 2 studies (online supplemental figure S6B). Most of the risk of bias was attributable to issues regarding attrition, randomisation and assessor blinding. The level of certainty of the evidence in the exercise studies of healthy and comorbid individuals was downgraded by one level due to limitations in study bias and heterogeneity, respectively (online supplemental table S3A). The level of certainty of the evidence in the caloric restriction studies was downgraded by one level due to limitations in study bias (online supplemental table S3B). We are moderately confident that the true effects are likely to be close to the estimates of the effects for both interventions.

### Meta-regression and subgroup analyses

Meta-regressions were performed to explore the potential influence of baseline characteristics. None of the chosen moderators was associated with the overall effects (online supplemental table S4A–S4B). Similarly, subgroup analyses were performed to explore potential variations in the ESs. A summary of the subgroup analyses performed is provided in online supplemental Table S5A–S5B.

### Sensitivity analyses

Leave-one-out diagnostics identified three influential exercise studies and one influential caloric restriction study. After removing the outliers, exercise produced an effect of −0.32 ((−0.41 to −0.23); p<0.001; I^2^=22%) and a dose–response effect of −0.14 ((−0.23 to −0.05); p=0.004) per 1000 calories deficit per week. The overall effect of caloric restriction became −0.46 ((−0.60 to −0.32); p<0.001; I^2^=18%), with a non-significant dose–response effect of −0.04 ((−0.17 to 0.08); p=0.49) per 1000 calories deficit per week.

After excluding the studies that did not report the prescribed caloric expenditures, the effects of exercise and caloric restriction interventions became −0.48 ((−0.69 to −0.27); p<0.001) and −0.59 ((−0.79 to −0.39); p<0.001), respectively. Consistent with the main results, exercise interventions revealed a dose–response effect of −0.16 ((−0.31 to −0.00); p=0.045) per 1000 calories deficit per week, while the effect of caloric restriction interventions was not dose-dependent (ES, 0.10 (−0.07 to 0.27); p=0.23).

We repeated the analyses after excluding studies with high risk of bias and the overall effect did not change for exercise (ES −0.27 (−0.38 to −0.17); p<0.001) and caloric restriction (ES −0.53 (−0.72 to −0.33); p<0.001). Similarly, there were no substantial differences changes in the dose–response effects of exercise (ES −0.13 (−0.22 to −0.03); p=0.009) and caloric restriction (ES 0.03 (−0.14 to 0.19); p=0.728).

## Discussion

This study aimed to determine the dose–response effectiveness of two lifestyle interventional strategies, physical exercise and caloric restriction, on reducing visceral fat in overweight and obese adults. Our findings support the notion that both interventions can effectively decrease the volume of visceral fat in this population; however, only exercise demonstrated a dose–dependent relationship with visceral fat. In contrast, both exercise and caloric restriction showed dose–response effects on reducing waist circumference.

To our knowledge, this is the first meta-analysis comparing the dose–response effects of exercise and caloric restriction by controlling for the weekly caloric deficit induced by the interventions. Our findings align with a previous meta-analysis comparing exercise and hypocaloric diets for visceral fat loss, which showed that exercise interventions induced greater reductions in visceral fat compared with caloric restriction.^16^ In the absence of weight loss, exercise produced a 6.1% reduction in visceral fat, whereas hypocaloric diets showed essentially no change.^16^ A study that randomised obese individuals to exercise or caloric restriction interventions with matching energy deficits found that participants in the exercise group had a two-fold greater reduction in visceral fat compared with the caloric restriction group.^35^ Similarly, Murphy *et al* reported a twofold greater loss of visceral adipose tissue in the exercise group compared with the caloric restriction group after adjusting for total fat changes in sedentary adults.^19^ However, several studies comparing exercise and caloric restriction within the same trial observed no differences in visceral fat changes between the two interventions,^36–39^ and several multiarm exercise studies involving interventions with different volumes or intensities failed to detect a dose–response relationship among the intervention groups.^40–42^ Under-reporting of caloric intake or overcompensating for the energy expended with excess food intake are common challenges in nutrition and metabolic research,^43 44^ which might explain the lack of differences in the treatment effects between groups. Overall, our results showed a dose-dependent effect of exercise on visceral fat, which was superior to the effect of caloric restriction. More evidence is warranted to elucidate the comparative effectiveness and the dose–dependent responses of these two lifestyle interventions.

### Clinical implications

Exercise and caloric restriction can both stimulate weight loss via a negative energy balance, which is achieved through an increased energy expenditure or a decreased caloric intake, respectively. Evidence shows that hypocaloric diets may be superior to exercise in achieving weight loss.^16 45^ This is likely because during caloric restriction both fat and muscle mass are reduced.^46 47^ On the other hand, exercise might stimulate fat loss while maintaining muscle mass.^48 49^ In fact, exercise-induced fat loss is achievable independently of weight loss.^35–37^ Research shows that metabolic adaptations to a low-calorie diet can differ among individuals, despite similar increases in fat oxidation rates, and that strong metabolic adaptations might mitigate the effect of caloric restriction on visceral fat.^50–54^ Conversely, previous literature emphasised the role of muscle mass in the regulation of resting energy expenditure.^55–57^ Our results suggest that exercise might be more suitable than caloric restriction for visceral fat loss in overweight and obese individuals. Differential metabolic adaptations and individual variations are potential causes for the difference in treatment responses to the two interventions.

Our results showed a dose–dependent effect for waist circumference in both exercise and caloric restriction interventions. These findings are promising, although they contrast with the primary outcome analyses, as visceral fat is strongly correlated with waist circumference,^58 59^ but our primary outcome analyses did not reveal a dose–response effect of caloric restriction on visceral fat. These findings align with a recent Consensus Statement by the International Atherosclerosis Society and the International Chair on Cardiometabolic Risk Working Group on Visceral Obesity, which described a plausible relationship between reductions in visceral fat and waist circumference, but concluded that a precise estimation of visceral fat from waist circumference is not possible.^60^ The relationship between visceral fat and anthropometric measurements such as waist circumference and BMI varies greatly among individuals in different age and sex groups.^61 62^ Therefore, it is not surprising that the large cohort analysed in this study showed differential responses to visceral fat and waist circumference outcomes. Taken together, our results support the dose–response effects of both exercise and caloric restriction strategies in reducing waist circumference in overweight and obese adults.

### Limitations

A limitation of this study was the disproportion between the number of exercise and caloric restriction studies. In fact, 46 and 16 effects were extrapolated from exercise and caloric restriction studies, respectively. This imbalance might have caused the lack of a significant dose–response effect for caloric restriction studies on visceral fat. Furthermore, four studies could not be included in the analyses due to missing data, which could not be obtained after contacting the respective authors. Similarly, information regarding caloric deficits or exercise intensities was at times lacking, and had to be calculated from the available data or requested from authors. Future research should adhere to validated reporting guidelines (eg, Consolidated Standards of Reporting Trials [CONSORT]) to facilitate the reporting and analysis of data. Lastly, the overall effect of exercise as well as its dose–response effect on visceral fat were small, which limits the interpretation of our results. Although the overall effect was increased after removing potential outliers and studies that did not report the prescribed caloric expenditure, the dose–response effect was essentially unchanged. Future studies should further explore the dose–response relationships of exercise and caloric restriction interventions on visceral fat to corroborate our findings.

## Conclusion

The findings of this study support the dose–dependent effects of exercise as an effective lifestyle interventional strategy to reduce visceral fat in overweight and obese adults. Caloric restriction did not demonstrate a dose–response relationship, although this may be attributed to the smaller number of studies available for analysis when compared with exercise studies. Secondary outcome analyses showed that both interventions produced dose–dependent responses on waist circumference. Further research is needed to elucidate the effects of caloric restriction on visceral fat.

## Data Availability

Data available upon request.
