# Supplementary material for: Dose–response effects of exercise and caloric restriction on visceral adiposity in overweight and obese adults: a systematic review and meta-analysis of randomised controlled trials
Source: Br J Sports Med. 2023 Jan 20;57(16):1035–41. doi: 10.1136/bjsports-2022-106304 (PMC10423480; doi:10.1136/bjsports-2022-106304)
Supplement: Supplementary data [file bjsports-2022-106304supp001.pdf]

Dose-Response Effects of Exercise and Caloric Restriction on Visceral Adiposity in Overweight and Obese Adults: A Systematic Review and Meta-Analysis of Randomized Controlled Trials

Supplementary Materials

Recchia F, *et al. Br J Sports Med* 2023; 57:1035–1043. doi: 10.1136/bjsports-2022-106304

Table of Contents

Table S1A Exercise intensity categorization (Reference)

Table S1B Exercise intensity categorization (Implementation)

Table S2A Additional study and intervention characteristics (exercise)

Table S2B Additional study and intervention characteristics (caloric restriction)

Table S3A Summary of findings for exercise (GRADE Framework)

Table S3B Summary of findings for caloric restriction (GRADE framework)

Table S4A Meta-regression analyses for exercise studies

Table S4B Meta-regression analyses for caloric restriction studies

Table S5A Subgroup analyses for exercise studies

Table S5B Subgroup analyses for caloric restriction studies

Fig. S1 PRISMA flow chart

Fig. S2 Forest plot of the effect of exercise on waist circumference

Fig. S3 Dose-response effect of exercise on waist circumference

Fig. S4 Forest plot of the effect of caloric restriction on waist circumference

Fig. S5 Dose-response effect of caloric restriction on waist circumference

Fig. S6A Risk of bias assessmment for exercise studies

Fig. S6B Risk of bias assessment for caloric restriction studies

Appendix S1 Search strategy

Appendix S2 Reference list

Recchia F, *et al. Br J Sports Med* 2023; 57:1035–1043. doi: 10.1136/bjsports-2022-106304

Table S1A Exercise intensity categorization (Reference)

Intensity	$\%HR_{reserve}$ $\%VO_{2reserve}$	$\%HR_{max}$	$\%VO_{2max}$	RPE 6-20	METs			%1-RM
					Young	Middle-aged	Older	
Very light (v. LIG)	< 30	< 57	< 37	≤ 9	< 2.4	< 2.0	< 1.6	< 30
Light (LIG)	30 – < 40	57 – < 64	37 – < 45	9 – 11	< 4.8	< 4.0	< 3.2	30 – < 50
Moderate (MOD)	40 – < 60	64 – < 76	46 – < 64	12 – 13	4.8 – < 7.2	4.0 – < 6.0	3.2 – < 4.8	50 – < 70
Vigorous (VIG)	60 – < 90	76 – < 96	64 – < 91	14 – 17	7.2 – < 10.2	6.0 – < 8.5	4.8 – < 6.8	70 – < 85
Near max to max (MAX)	≥ 90	≥ 96	≥ 91	≥ 18	≥ 10.2	≥ 8.5	≥ 6.8	≥ 85

Adopted from the American College of Sports Medicine's Guidelines for Exercise Testing and Prescription (9th Edition).
1-RM One-Repetition Maximum; METs Metabolic Equivalent of Task; RPE Rate of Perceived Exertion.

Recchia F, *et al. Br J Sports Med* 2023; 57:1035–1043. doi: 10.1136/bjsports-2022-106304

Table S1B Exercise intensity categorization (Implementation)

Study	Groups	Prescribed Intensity	Categorization
Abdelbasset et al., 2019, 2020a,b	HIIT (1)	80-85% VO_{2max}<br>50% VO_{2max}	**VIG**
	MICT (2)	60-70% HR_{max}	**LIG – MOD**
Blond et al., 2019	MOD (1) (2)	50% $VO_{2reserve}$	**MOD**
	VIG (3) (4)	70% $VO_{2reserve}$	**VIG**
Cho et al., 2011	HI (1)	70-75% VO_{2max}	**VIG**
	LI (2)	40-50% VO_{2max}	**LIG – MOD**
Coker et al., 2009a	EX	50% VO_{2peak}	**MOD**
Cowan et al., 2018	LILV (1)	50% VO_{2peak}	**MOD**
	LIHV (2)	50% VO_{2peak}	**MOD**
	HIHV (3)	75% VO_{2peak}	**VIG**
Hallsworth et al., 2015	HIIT	RPE 16 – 17<br>NA	**VIG**
Hong et al., 2014	EX	50-60% VO_{2max}	**MOD**
Irving et al., 2008, 2009	HI (1)	RPE 15 – 17<br>RPE 10 – 12	**LIG – VIG**<br>(taken as **MOD**)
	LI (2)	RPE 10 – 12	**LIG – MOD**
Johnson et al., 2009	EX	50-70% VO_{2peak}	**MOD – VIG**
Jung et al., 2012	MOD (1)	3.5-5.2 METs	**LIG – MOD**
	VIG (2)	> 5.3 METs	**MOD – VIG**
Keating et al., 2015	HILV (1)	70% VO_{2peak}<br>50% VO_{2peak}	**VIG**<br>**MOD**

Recchia F, *et al. Br J Sports Med* 2023; 57:1035–1043. doi: 10.1136/bjsports-2022-106304

	LIHV (2)<br>LILV (3)	50% VO_{2peak}	**MOD**
Keating et al., 2017	RT	80-85% 1-RM	**VIG**
Lesser et al., 2016	EX	55-85% HR_{max}	**LIG – VIG**<br>(taken as **MOD**)
Nordby et al., 2012<br>Bladbjerg et al., 2017	EX	65-85% $HR_{reserve}$	**VIG**
Reichkendler et al., 2013	HV (1)<br>MV (2)	> 70% VO_{2max}<br>50-70% VO_{2max}<br>> 70% VO_{2max}<br>50-70% VO_{2max}	**MOD – VIG**<br>**MOD – VIG**
Ross et al., 2000<br>Thong et al., 2000	EX	≤ 70% VO_{2peak}	**MOD – VIG**
Ross et al., 2004	EX	80% HR_{max}	**VIG**
Saremi et al., 2010	EX	60-85% HR_{max}	**LIG – VIG**<br>(taken as **MOD**)
Shojaee-Moradie et al., 2007	EX	60-85% VO_{2max}	**MOD – VIG**
Slentz et al., 2005	HIHV<br>HILV<br>MILV	65-80% VO_{2peak}<br>65-80% VO_{2peak}<br>40-55% VO_{2peak}	**VIG**<br>**VIG**<br>**LIG – MOD**
Wu et al., 2017	HI (1) (2)<br>LI (3) (4)	65-70% VO_{2max}<br>50-56% VO_{2max}	**VIG**<br>**MOD**
Zhang et al., 2015	HIIT (1)<br>MICT (2)	85-95% HR_{peak}<br>50-60% HR<br>60-70% HR_{peak}	**VIG**<br>**MOD**
Zhang et al., 2016	MOD (1)<br>VIG (2)	3.0 – 6.0 METs<br>8.0 – 10.0 METs	**LIG – MOD**<br>**VIG – MAX**

Recchia F, *et al. Br J Sports Med* 2023; 57:1035–1043. doi: 10.1136/bjsports-2022-106304

Zhang et al., 2017	HIIT (1)	90% VO_{2max} Passive	**VIG**
	MICT (2)	60% VO_{2max}	**MOD**

EX exercise; **HI** high intensity; **HIIT** High-intensity interval training; **HV** high volume; **LI** low intensity; **LIG** light intensity; **LV** low volume; **MI** moderate intensity; **MICT** Moderate intensity continuous training; **MOD** moderate intensity; **MV** moderate volume; **RT** resistance training; **VIG** vigorous intensity.

Table S2A Additional study and intervention characteristics (exercise)

Study	Location	Groups[a]	Frequency	Intensity	Time	Caloric deficit
Abdelbasset et al., 2019, 2020a,b	Egypt	HIIT (1)	3 d/wk	80%-85% VO_{2max} & 50%VO_{2max}	3 x 4 min & 2 x 2 min	NR
		MICT (2)	3 d/wk	60%-70% HR_{max}	40-50 min	NR
Blond et al., 2019	Denmark	MOD (1)	5 d/wk	50% $VO_{2reserve}$	NR	370 kcal/d
		VIG (2)	5 d/wk	70% $VO_{2reserve}$	NR	370 kcal/d
Cho et al., 2011	South Korea	HI (1)	3 d/wk	70%-75% VO_{2max}	NR	400 kcal/d
		LI (2)	3 d/wk	40%-50% VO_{2max}	NR	400 kcal/d
Coker et al., 2009a	United States	AE	NR	50% VO_{2peak}	NR	2,500 kcal/wk
Coker et al., 2009b[*]	United States	HI (1)	4-5 d/wk	75% VO_{2peak}	NR	1,000 kcal/wk
		MI (2)	4-5 d/wk	50% VO_{2peak}	NR	1,000 kcal/wk
Cowan et al., 2018	Canada	LILV (1)	5 d/wk	50%VO_{2peak}	NR	240 kcal/d
		LIHV (2)	5 d/wk	50%VO_{2peak}	NR	480 kcal/d
		HIHV (3)	5 d/wk	75%VO_{2peak}	NR	480 kcal/d
Davidson et al., 2009[*]	Canada	AE	5 d/wk	60%-75% VO_{2peak}	30 min	NR
Hallsworth et al., 2015	United Kingdom	HIIT	3 d/wk	RPE 16 – 17	30-40 min	NR
Hong et al., 2014	South Korea	AE	3 d/wk	50%-60% VO_{2max}	50-70 min	400 kcal/d
Irving et al., 2008, 2009	United States	HI (1)	5 d/wk	RPE 15 – 17	NR	400 kcal/d
		LI (2)	2 d/wk	RPE 10 – 12	NR	400 kcal/d
Johnson et al., 2009	Australia	AE	3 d/wk	50%-70% VO_{2peak}	30-45 min	280 kcal/d
Jung et al., 2012	South Korea	MOD (1)	5 d/wk	3.5-5.2 METs	60 min	500 kcal/d
		VIG (2)	5 d/wk	> 5.3 METs	30 min	500 kcal/d
Keating et al., 2015	Australia	HILV (1)	3 d/wk	70% VO_{2peak}	45 min	360 kcal/d
		LIHV (2)	4 d/wk	50% VO_{2peak}	60 min	360 kcal/d

Recchia F, *et al. Br J Sports Med* 2023; 57:1035–1043. doi: 10.1136/bjsports-2022-106304

		LILV (3)	3 d/wk	50% VO_{2peak}	45 min	220 kcal/d
Keating et al., 2017	Australia	RT	3 d/wk	80%-85% 1-RM	30-60 min	NR
Koo et al., 2010	South Korea	AE	7 d/wk	NR	120 min	500 kcal/d
Lee et al., 2012*	South Korea	HI (1)<br>LI (2)	3-5 d/wk<br>3-5 d/wk	70% VO_{2max}<br>50% VO_{2max}	NR<br>NR	13.5-22.5 METs-h/wk<br>13.5-22.5 METs-h/wk
Lesser et al., 2016	Canada	AE	3 d/wk	55%-85% HR_{max}	40 min	NA
Nordby et al., 2012 Bladbjerg et al., 2017	Denmark	AE	7 d/wk	65%/85% $HR_{reserve}$	NR	600 kcal/d
Pugh et al., 2014* Cuthbertson et al., 2016*	United Kingdom	AE	3-5 d/wk	30%-60% $HR_{reserve}$	30-45 min	230 kcal/d
Reichkendler et al., 2013	Denmark	HV (1)<br>MV (2)	3 d/wk<br>4 d/wk	> 70% VO_{2max}<br>50%-70% VO_{2max}	NR<br>NR	600 kcal/d<br>300 kcal/d
Ross et al., 2000 Thong et al., 2000	Canada	AE	7 d/wk	≤ 70% VO_{2peak}	NR	700 kcal/d
Ross et al., 2004	Canada	AE	7 d/wk	80% HR_{max}	NR	500 kcal/d
Saremi et al., 2010	Iran	AE	5 d/wk	60%-85% HR_{max}	15-50 min	NR
Schmitz et al., 2007	United States	RT	2 d/wk	NR	45-60 min	NR
Shojaee-Moradie et al., 2007	United Kingdom	AE	3 d/wk	60%-85% VO_{2max}	20 min	200 kcal/d
Slentz et al., 2005	United States	HIHV (1)<br>HILV (2)<br>MILV (3)	NR<br>NR<br>NR	65%-80% VO_{2peak}<br>65%-80% VO_{2peak}<br>40%-55% VO_{2peak}	NR<br>NR<br>NR	23 kcal/kg/wk<br>14 kcal/kg/wk<br>14 kcal/kg/wk
Wu et al., 2017	South Korea & United States	HI (1)<br>LI (2)	4-5 d/wk<br>4-5 d/wk	65%-70% VO_{2max}<br>50%-56% VO_{2max}	NR<br>NR	13.5-22.5 METs-h/wk<br>13.5-22.5 METs-h/wk
Zhang et al., 2015	China	HIIT (1)	4 d/wk	90% HR_{peak} & 55%HR_{peak}	4 x 4min & 3 x 3min	249 kcal/d

		MICT (2)	4 d/wk	65% HR_{peak}	33min	253 kcal/d
Zhang et al., 2016	China	MOD (1)	5 d/wk	3.0 – 6.0 METs	30 min	170 kcal/d
		VIG (2)	5 d/wk	8.0 – 10.0 METs	30 min	340 kcal/d
Zhang et al., 2017	China	HIIT (1)	3-4 d/wk	90% VO_{2max} Passive	NR	60 kcal/d
		MICT (2)	3-4 d/wk	60% VO_{2max}	NR	60 kcal/d

AE aerobic exercise; **HI** high intensity; **HIIT** high-intensity interval training; **HR_{max}** maximum heart rate; **$HR_{reserve}$** heart rate reserve; **HV** high volume; **LI** low intensity; **LV** low volume; **METs** metabolic equivalents of task; **MI/MOD** moderate intensity; **MICT** moderate-intensity continuous training; **MV** moderate volume; **NR** not reported; **RM** repetition maximum; **RPE** rating of perceived exertion; **RT** resistance training; **VIG** vigorous intensity; **VO_{2max}** maximal oxygen uptake; **VO_{2peak}** peak oxygen uptake; **$VO_{2reserve}$** oxygen uptake reserve

* NOT included in the meta-analysis due to insufficient data
[a]Intervention arms being synthesized only

Table S2B Additional study and intervention characteristics (caloric restriction)

Study	Location	Groups[a]	Diet Prescription	Caloric deficit
Bouchonville et al., 2014<br>Napoli et al., 2014	United States	CR	NR	500-750 kcal/d
Brennan et al., 2021	United States	CR	NR	500-1000 kcal/d
Coker et al., 2009a	United States	CR	NR	2,500 kcal/wk
Ibáñez et al., 2010<br>Idoate et al., 2011<br>García-Unciti et al., 2012	Spain	WL	NR	500 kcal/d
Kang et al., 2018	South Korea	LCD	NR	300 kcal/d
Koo et al., 2010	South Korea	CR	1200 kcal/d	550 kcal/d
Larson-Meyer et al., 2006, 2010<br>Redman et al., 2007, 2010	United States	CR	25% daily deficit	700 kcal/d
Lee et al., 2018	South Korea	WL	NR	300 kcal/d
Ng et al., 2007, 2009<br>Chan et al., 2008	Australia	WL	6,143 kJ/d	870 kcal/d
Nordby et al., 2012<br>Bladbjerg et al., 2017	Denmark	CR	NR	600 kcal/d
Ross et al., 2000<br>Thong et al., 2000	Canada	CR	NR	700 kcal/d
Ross et al., 2004	Canada	WL	NR	500 kcal/d
Schübel et al., 2018	Germany	CR	20% daily deficit	410 kcal/d
Schutte et al., 2022	The Netherlands	LNCR (1)<br>HNCR (2)	25% daily deficit<br>25% daily deficit	415 kcal/d<br>415 kcal/d
Trepanowski et al., 2018	United States	CR	25% daily deficit	730 kcal/d

CR caloric restriction; **HN** High nutrient; **LCD** low-calorie diet; **LN** Low nutrient; **NR** not reported; **WL** weight loss; **WM** weight maintenance

[a]Intervention arms being synthesized only

Recchia F, *et al. Br J Sports Med* 2023; 57:1035–1043. doi: 10.1136/bjsports-2022-106304

Table S3A Summary of findings for exercise (GRADE Framework)

**Effect of exercise in adults with excess adiposity**			
**Population:** Adults (≥ 18 years old) with an elevated BMI and/or waist circumference<br>**Setting:** Community/outpatient<br>**Interventions:** Exercise<br>**Comparisons:** Usual care/no intervention/health education/weight maintenance			
**Outcomes**	**Effect size (95% CI)**	**Participants (Studies)**	**Quality of Evidence (GRADE)***
Visceral fat (healthy)	-0.34 (-0.45 to -0.23)	921 (19)	⊕⊕⊕⊖[a]
Visceral fat (comorbidities)	-0.17 (-0.33 to -0.01)	431 (7)	⊕⊕⊕⊖[b]

*Certainty of evidence grades:
(1) ⊕⊕⊕⊕ High: further research is unlikely to change the confidence in the estimate of effect.
(2) ⊕⊕⊕⊖ Moderate: further research is likely to have an important impact on the confidence in the estimate of effect and may change the estimate.
(3) ⊕⊕⊖⊖ Low: further research is very likely to have an important impact on the confidence in the effect estimate and is likely to change the estimate.
(4) ⊕⊖⊖⊖ Very low: any estimate of effect is very uncertain.

[a]Downgraded by one level for risk of bias: most of the included studies were susceptible to at least moderate risk of bias.
[b]Downgraded by one level for heterogeneity: compared to the healthy counterparts, the heterogeneity is higher among studies of participants with comorbidities. Nonetheless, more than half of the studies were only susceptible to low risk of bias. The certainty of evidence was therefore downgraded by one level.

Recchia F, *et al. Br J Sports Med* 2023; 57:1035–1043. doi: 10.1136/bjsports-2022-106304

Table S3B Summary of findings for caloric restriction (GRADE Framework)

**Effect of caloric restriction in adults with excess adiposity**			
**Population:** Adults (≥ 18 years old) with an elevated BMI and/or waist circumference<br>**Setting:** Community/outpatient<br>**Interventions:** Caloric restriction<br>**Comparisons:** Usual care/no intervention/health education/weight maintenance			
**Outcomes**	**Effect size (95% CI)**	**Participants (Studies)**	**Quality of Evidence (GRADE)***
Visceral fat	-0.53 (-0.71 to -0.35)	721 (15)	⊕⊕⊕⊖[a]

*Certainty of evidence grades:
(1) ⊕⊕⊕⊕ High: further research is unlikely to change the confidence in the estimate of effect.
(2) ⊕⊕⊕⊖ Moderate: further research is likely to have an important impact on the confidence in the estimate of effect and may change the estimate.
(3) ⊕⊕⊖⊖ Low: further research is very likely to have an important impact on the confidence in the effect estimate and is likely to change the estimate.
(4) ⊕⊖⊖⊖ Very low: any estimate of effect is very uncertain.

[a]Downgraded by one level for risk of bias: most of the included studies were susceptible to at least moderate risk of bias.

Table S4A Meta-regression analyses for exercise studies

**Moderator**	**K**	**Effect [95% CI]**	**P value**
Intervention duration	46	.00 [-.00 to .00]	.95
Age	41	.01 [.00 to .02]	.14
BMI	42	-.03 [-.07 to .00]	.05
Sex	44	-.18 [-.44 to .08]	.16
Exercise frequency (continuous)	42	-.02 [-.08 to .05]	.66

Recchia F, *et al. Br J Sports Med* 2023; 57:1035–1043. doi: 10.1136/bjsports-2022-106304

Table S4B Meta-regression analyses for caloric restriction studies

Moderator	K	Effect [95% CI]	P value
Intervention duration	16	.00 [-.01 to .02]	.69
Age	15	.00 [-.02 to .02]	.79
BMI	16	.01 [-.05 to .06]	.78
Sex	15	-.37 [-.98 to .23]	.21

Recchia F, *et al. Br J Sports Med* 2023; 57:1035–1043. doi: 10.1136/bjsports-2022-106304

Table S5A Subgroup analyses for exercise studies

Moderator	K	Effect [95% CI]	P value
**Exercise frequency (categorical)**			
≤ 3.5 d/wk	15	-.31 [-.48 to -.14]	< .001
> 3.5 d/wk	22	-.25 [-.40 to -.09]	= .002
**Intensity**			
Low	6	-.11 [-.33 to .11]	= .11
Moderate	22	-.32 [-.46 to -.17]	< .001
Vigorous	16	-.34 [-.49 to -.19]	< .001
**Disease**			
Healthy	33	-.34 [-.45 to -.23]	< .001
DM	6	-.21 [-.48 to .05]	= .11
METS	2	-.02 [-.49 to .45]	= .92
NAFLD	5	-.18 [-.39 to .04]	= .10
**Measure**			
MRI	20	-.22 [-.32 to -.11]	< .001
CT	26	-.44 [-.57 to -.30]	< .001
**Supervision**			
Supervised	35	-.33 [-.43 to -.22]	< .001
Unsupervised	11	-.18 [-.36 to .00]	= .05
**Continent**			
Asia	19	-.23 [-.40 to -.06]	= .012
USA	13	-.30 [-.46 to -.15]	= .001
Europe	9	-.45 [-.75 to -.14]	= .010
Australia	5	-.22 [-.35 to -.09]	= .009

CT Computed Tomography; DM Diabetes Mellitus; METS Metabolic Syndrome; MRI Magnetic Resonance Imaging; NAFLD Non-Alcoholic Fatty Liver Disease.

Table S5B Subgroup analyses for caloric restriction studies

Moderator	K	Effect [95% CI]	P value
**Disease**			
Healthy	14	-.55 [-.73 to -.36]	< .001
DM	1	-.06 [-.86 – .78]	= .86
METS	1	-.07 [-1.50 to .05]	= .05
**Measure**			
MRI	11	-.50 [-.73 to -.28]	= .002
CT	5	-.58 [-.91 to -.25]	< .001
**Continent**			
Asia	3	-.57 [-2.19 to 1.04]	= .27
USA	7	-.50 [-.61 to -.38]	< .001
Europe	5	-.50 [-.95 to -.06]	= .034
Australia	1	-.74 [-1.44 to -.04]	= .038

CT Computed Tomography; DM Diabetes Mellitus; METS Metabolic Syndrome; MRI Magnetic Resonance Imaging.

Fig. S1 PRISMA Flow chart

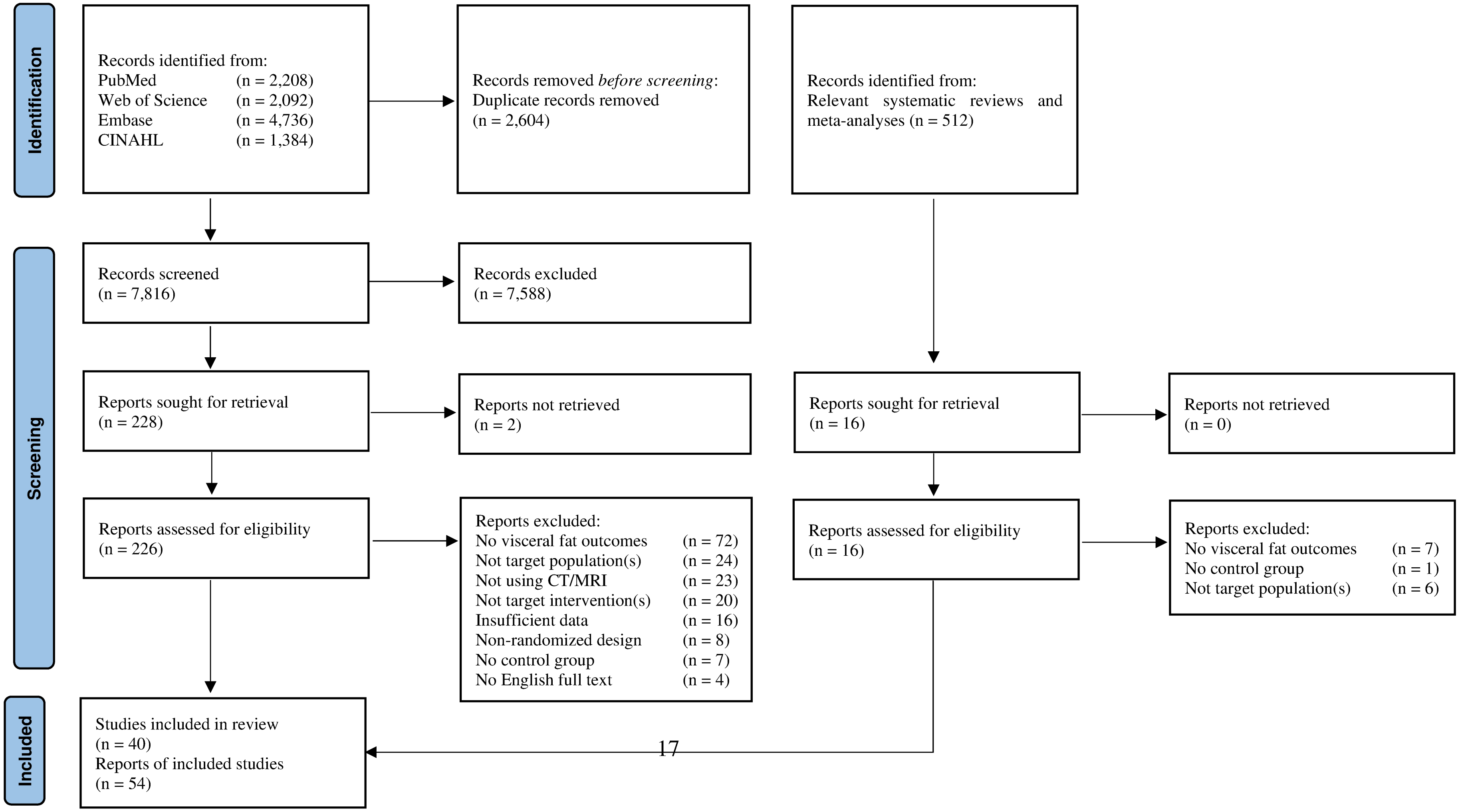

Fig. S2 Forest plot of the effect of exercise on waist circumference

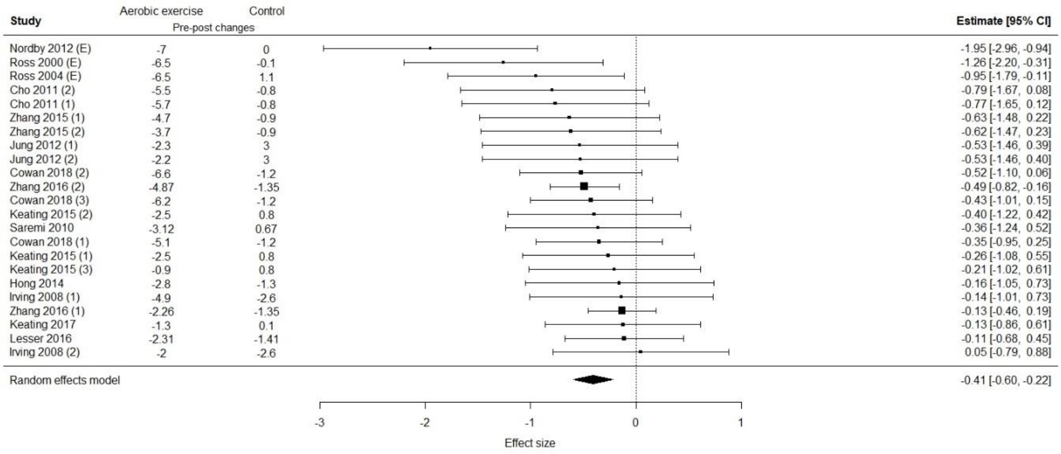

Fig. S3 Dose-response effect of exercise on waist circumference

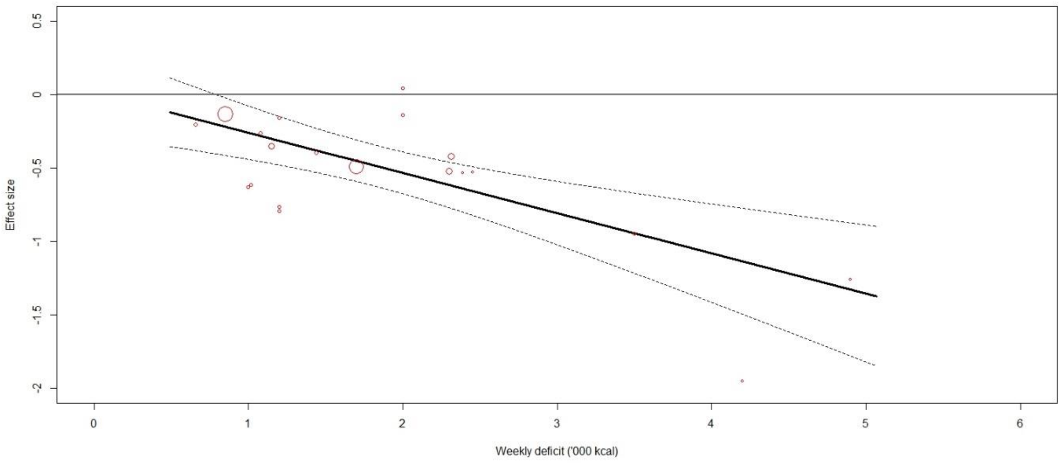

Recchia F, *et al. Br J Sports Med* 2023; 57:1035–1043. doi: 10.1136/bjsports-2022-106304

Fig. S4 Forest plot of the effect of caloric restriction on waist circumference

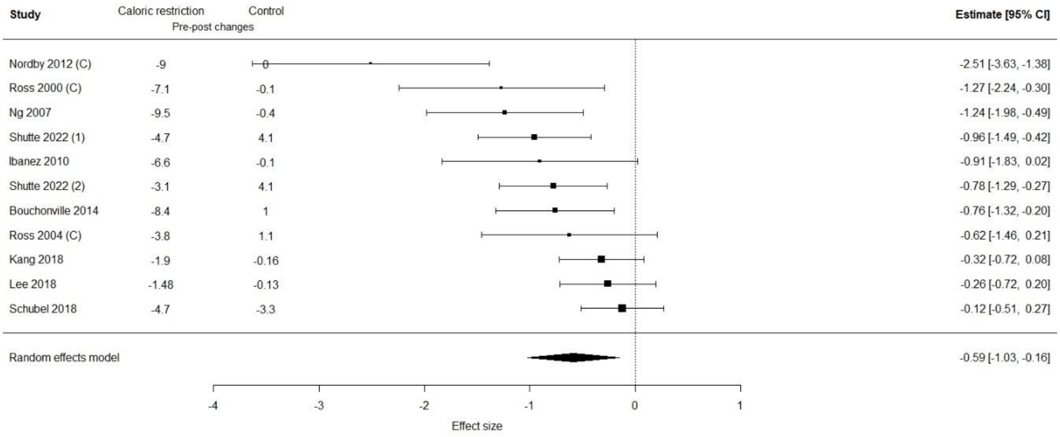

Fig. S5 Dose-response effect of caloric restriction on waist circumference

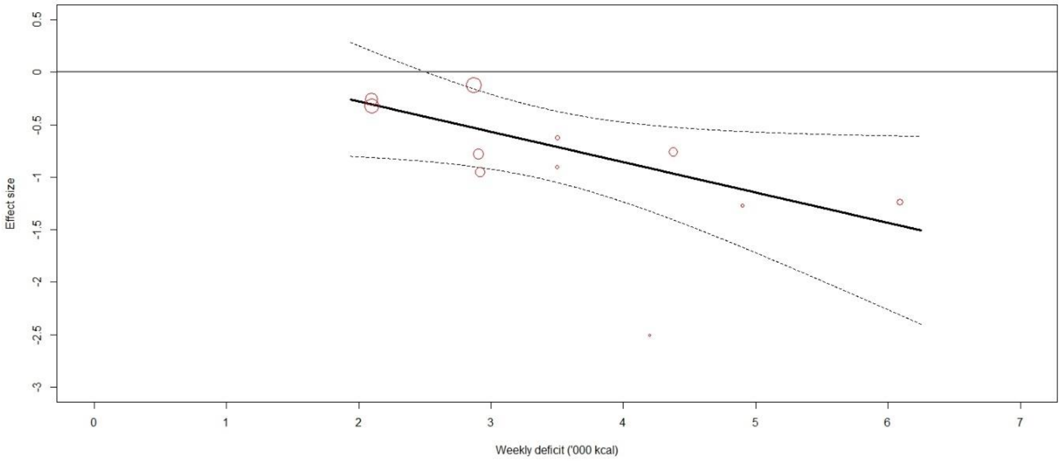

Recchia F, *et al. Br J Sports Med* 2023; 57:1035–1043. doi: 10.1136/bjsports-2022-106304

Fig. S6A Risk of Bias assessmment for exercise studies

Study ID	D1	D2	D3	D4	D5	Overall
Abdelbasset 2020	+	+	+	+	+	+
Blond 2019	+	!	+	+	+	!
Cho 2011	+	-	+	+	+	-
Coker 2009a	!	-	+	!	+	-
Cowan 2018	+	+	!	!	+	!
Hallsworth 2015	+	+	+	!	+	!
Hong 2014	!	+	+	!	+	!
Irving 2008	!	!	+	+	+	!
Johnson 2009	+	+	+	+	+	+
Jung 2012	!	-	+	+	+	-
Keating 2015	+	+	+	+	+	+
Keating 2017	+	+	+	+	+	+
Koo 2010	!	+	+	!	+	!
Lesser 2016	+	+	+	+	+	+
Nordby 2012	+	-	+	+	+	-
Reichkendler 2013	!	+	+	+	+	!
Ross 2000	+	+	+	!	+	!
Ross 2004	!	+	!	!	+	!
Saremi 2010	!	+	+	!	+	!
Schmitz 2007	+	!	!	+	+	!
Shojaee-Moradie 2007	!	+	+	!	+	!
Slentz 2005	!	-	+	!	+	-
Wu 2017	!	+	+	+	+	!
Zhang 2015	!	+	+	+	+	!
Zhang 2016	+	+	+	+	+	+
Zhang 2017	!	+	+	+	+	!

+ Low risk
! Some concerns
- High risk

D1 Randomisation process
D2 Deviations from the intended interventions
D3 Missing outcome data
D4 Measurement of the outcome
D5 Selection of the reported result

Recchia F, *et al. Br J Sports Med* 2023; 57:1035–1043. doi: 10.1136/bjsports-2022-106304

Fig. S6B Risk of Bias assessment for caloric restriction studies

Study ID	D1	D2	D3	D4	D5	Overall
Bouchonville 2014	+	+	+	+	+	+
Brennan 2021	+	!	+	+	+	!
Coker 2009a	!	-	+	!	+	-
Ibáñez 2010	!	+	+	!	+	!
Kang 2018	+	+	+	!	+	!
Koo 2010	!	+	+	!	+	!
Larson-Meyer 2006	!	+	+	+	+	!
Lee 2018	+	+	+	!	+	!
Ng 2007	!	+	+	!	+	!
Nordby 2012	+	-	+	+	+	-
Ross 2000	+	+	+	!	+	!
Ross 2004	!	+	!	!	+	!
Schübel 2018	+	+	+	+	+	+
Shutte 2022	+	+	+	+	+	+
Trepanowski 2018	!	+	+	!	+	!

+ Low risk

! Some concerns

- High risk

D1 Randomisation process

D2 Deviations from the intended interventions

D3 Missing outcome data

D4 Measurement of the outcome

D5 Selection of the reported result

Recchia F, *et al. Br J Sports Med* 2023; 57:1035–1043. doi: 10.1136/bjsports-2022-106304

Appendix S1 Search strategy

PubMed

(overweight OR obesity OR central obesity) AND (exercise OR "physical training" OR endurance training OR "aerobic training" OR "continuous training" OR resistance training OR "strength training" OR "weight training" OR "interval training" OR "intermittent training" OR "HIIT" OR caloric restriction OR calori* restrict* OR calori* reduc* OR "low calorie diet*" OR "hypocaloric diet*" OR diet, reducing[MH] OR "diet intervention*" OR "dietary intervention*" OR "energy restrict*" OR "low energy diet*" OR weight loss[MH] OR "weight reduction") AND (visceral fat OR intra abdominal fat OR abdominal fat OR adipose tissue) AND ((randomized controlled trial[PT] OR controlled clinical trial[PT] OR randomized[TIAB] OR randomised[TIAB] OR placebo[TIAB] OR randomly[TIAB] OR trial[TIAB] OR groups[TIAB]) NOT (animals[MH] NOT humans[MH]))

Web of Science

(ALL=(overweight OR obes* OR "central* obes*" OR "abdominal* obes*")) AND (ALL=(exercise OR "physical training" OR "endurance training" OR "aerobic training" OR "continuous training" OR "resistance training" OR "strength training" OR "weight training" OR "interval training" OR "intermittent training" OR "HIIT" OR "calori* restrict*" OR "calori* reduc*" OR "low calorie" OR hypocaloric OR diet$ OR "diet* intervention*" OR "diet* restrict*" OR "energy restrict*" OR "energy reduc*" OR "weight loss" OR "weight reduction")) AND (ALL=("CON" OR control* OR "usual care" OR "conventional care" OR "standard care" OR "no intervention" OR "health education")) AND ((ALL=(visceral OR abdominal OR intraabdominal)) AND (ALL=(fat OR "adipose tissue" OR adiposity OR lipid$))) AND (TI=(randomized OR randomised OR randomly OR placebo OR trial OR groups) OR AB=(randomized OR randomised OR randomly OR placebo OR trial OR groups))

Embase

((exp overweight/ or overweight or exp obesity/ or obes* or ((abdominal* or central*) adj obes*)) and (exercise or ((physical or endurance or aerobic or continuous or resistance or strength or weight or interval or intermittent) adj training) or HIIT or (calori* adj (restrict* or reduc*)) or low calor* diet* or hypocaloric diet* or (diet* adj

(intervention* or restrict* or therapy)) or energy restrict* or low energy diet* or (weight adj (loss or reduction))) and ((visceral fat or intraabdominal fat or abdominal fat or adipose tissue))).mp. and ((randomized controlled trial/ or controlled clinical trial/ or controlled study/ or (randomized or randomised or placebo or randomly or trial or groups).ti,ab.) not ((animal/ or nonhuman/) not human/))

CINAHL

Search modes: Boolean/Phrase; Expanders: Also search within the full text of the articles

(overweight OR obes* OR (MH "obesity+") OR "central* obes*" OR "abdominal* obes*") AND (exercise OR (MH "exercise+") OR "physical training" OR "endurance training" OR "aerobic training" OR "continuous training" OR "resistance training" OR "strength training" OR "weight training" OR "interval training" OR "intermittent training" OR "HIIT" OR (calori* W1 (restrict* OR reduc*)) OR "low calorie diet*" OR "hypocaloric diet*" OR (MH "diet, reducing") OR (MH "restricted diet") OR "diet* restrict*" OR "diet* intervention*" OR "energy restrict*" OR "low energy diet*" OR (weight W1 (loss OR reduction))) AND (((visceral OR intraabdominal OR abdominal) W1 fat) OR adipose tissue) AND ((PT randomized controlled trial OR (MH "randomized controlled trial") OR PT clinical trial OR (MH "clinical trial") OR TI (randomi#ed OR placebo OR randomly OR trial OR groups) OR AB (randomi#ed OR placebo OR randomly OR trial OR groups)) NOT ((MH "animals") NOT (MH "human")))

Recchia F, *et al. Br J Sports Med* 2023; 57:1035–1043. doi: 10.1136/bjsports-2022-106304

Appendix S2 Reference list

Abdelbasset et al., 2019, 2020a,b

Abdelbasset WK, Tantawy SA, Kamel DM, Alqahtani BA, Soliman GS. A randomized controlled trial on the effectiveness of 8-week high-intensity interval exercise on intrahepatic triglycerides, visceral lipids, and health-related quality of life in diabetic obese patients with nonalcoholic fatty liver disease. *Medicine*. 2019;98(12):e14918.

Abdelbasset WK, Elsayed SH, Nambi G, Alrawaili SM, Elnegamy TE, Khalil MA, et al. Effect of moderate-intensity aerobic exercise on hepatic fat content and visceral lipids in hepatic patients with diabesity: a single-blinded randomised controlled trial. *Evid Based Complement Alternat Med*. 2020;2020:1923575.

Abdelbasset WK, Tantawy SA, Kamel DM, Alqahtani BA, Elnegamy TE, Soliman GS, et al. Effects of high-intensity interval and moderate-intensity continuous aerobic exercise on diabetic obese patients with nonalcoholic fatty liver disease: a comparative randomized controlled trial. *Medicine*. 2020;99(10):e19471.

Blond MB, Rosenkilde M, Gram AS, Tindborg M, Christensen AN, Quist JS, et al. How does 6 months of active bike commuting or leisure-time exercise affect insulin sensitivity, cardiorespiratory fitness and intra-abdominal fat? A randomised controlled trial in individuals with overweight and obesity. *Br J Sports Med*. 2019;53(18):1183-92.

Bouchonville et al., 2014; Napoli et al., 2014

Bouchonville M, Armamento-Villareal R, Shah K, Napoli N, Sinacore DR, Qualls C, et al. Weight loss, exercise or both and cardiometabolic risk factors in obese older adults: results of a randomized controlled trial. *Int J Obes*. 2014;38(3):423-31.

Napoli N, Shah K, Waters DL, Sinacore DR, Qualls C, Villareal DT. Effect of weight loss, exercise, or both on cognition and quality of life in obese older adults. *Am J Clin Nutr*. 2014;100(1):189-98.

Brennan AM, Standley RA, Anthony SJ, et al. Weight loss and exercise differentially affect insulin sensitivity, body composition, cardiorespiratory fitness and muscle strength in older adults with obesity; a randomized controlled trial. *J Gerontol A Biol Sci Med Sci*. Aug 18 2021;doi:10.1093/gerona/glab240

Cho JK, Lee SH, Lee JY, Kang HS. Randomized controlled trial of training intensity in adiposity. *Int J Sports Med*. 2011;32(6):468-75.

Coker RH, Williams RH, Kortebein PM, Sullivan DH, Evans WJ. Influence of exercise intensity on abdominal fat and adiponectin in elderly adults. *Metab Syndr Relat Disord*. 2009;7(4):363-8.

Coker RH, Williams RH, Yeo SE, Kortebein PM, Bodenner DL, Kern PA, et al. The impact of exercise training compared to caloric restriction on hepatic and peripheral insulin resistance in obesity. *J Clin Endocrinol Metab*. 2009;94(11):4258-66.

Cowan TE, Brennan AM, Stotz PJ, Clarke J, Lamarche B, Ross R. Separate Effects of Exercise Amount and Intensity on Adipose Tissue and Skeletal Muscle Mass in Adults with Abdominal Obesity. *Obesity*. 2018;26(11):1696-1703. doi:https://doi.org/10.1002/oby.22304

Davidson LE, Hudson R, Kilpatrick K, Kuk JL, McMillan K, Janiszewski PM, et al. Effects of exercise modality on insulin resistance and functional limitation in older adults: a randomized controlled trial. *Arch Intern Med*. 2009;169(2):122-31.

Hallsworth K, Thoma C, Hollingsworth KG, Cassidy S, Anstee QM, Day CP, et al. Modified high-intensity interval training reduces liver fat and improves cardiac function in non-alcoholic fatty liver disease: a randomized controlled trial. *Clin Sci*. 2015;129(12):1097-105.

Hong HR, Jeong JO, Kong JY, Lee SH, Yang SH, Ha CD, et al. Effect of walking exercise on abdominal fat, insulin resistance and serum cytokines in obese women. *J Exerc Nutr Biochem*. 2014;18(3):277-85.

Ibáñez et al., 2010; Idoate et al., 2011; García-Unciti et al., 2012

Ibáñez J, Izquierdo M, Martínez-Labari C, Ortega F, Grijalba A, Forga L, et al. Resistance training improves cardiovascular risk factors in obese women despite a significative decrease in serum adiponectin levels. *Obesity*. 2010;18(3):535-41.

Idoate F, Ibañez J, Gorostiaga EM, García-Unciti M, Martínez-Labari C, Izquierdo M. Weight-loss diet alone or combined with resistance training induces different regional visceral fat changes in obese women. *Int J Obes*. 2011;35(5):700-13.

García-Unciti M, Izquierdo M, Idoate F, Gorostiaga E, Grijalba A, Ortega-Delgado F, et al. Weight-loss diet alone or combined with progressive resistance training induces changes in association between the cardiometabolic risk profile and abdominal fat depots. *Ann Nutr Metab*. 2012;61(4):296-304.

Irving et al., 2008, 2009

Irving BA, Davis CK, Brock DW, Weltman JY, Swift D, Barrett EJ, et al. Effect of exercise training intensity on abdominal visceral fat and body composition. *Med Sci Sports Exerc*. 2008;40(11):1863-72.

Irving BA, Weltman JY, Patrie JT, Davis CK, Brock DW, Swift D, et al. Effects of exercise training intensity on nocturnal growth hormone secretion in obese adults with the metabolic syndrome. *J Clin Endocrinol Metab*. 2009;94(6):1979-86.

Johnson NA, Sachinwalla T, Walton DW, Smith K, Armstrong A, Thompson MW, et al. Aerobic exercise training reduces hepatic and visceral lipids in obese individuals without weight loss. *Hepatology*. 2009;50(4):1105-12.

Jung JY, Han KA, Ahn HJ, Kwon HR, Lee JH, Park KS, et al. Effects of aerobic exercise intensity on abdominal and thigh adipose tissue and skeletal muscle attenuation in overweight women with type 2 diabetes mellitus. *Diabetes Metab J*. 2012;36(3):211-21.

Kang M, Yoo HJ, Kim M, Kim M, Lee JH. Metabolomics identifies increases in the acylcarnitine profiles in the plasma of overweight subjects in response to mild weight loss: a randomized, controlled design study. *Lipids Health Dis*. 2018;17(1):237.

Keating SE, Hackett DA, Parker HM, O'Connor HT, Gerofi JA, Sainsbury A, et al. Effect of aerobic exercise training dose on liver fat and visceral adiposity. *J Hepatol*. 2015;63(1):174-82.

Keating SE, Hackett DA, Parker HM, Way KL, O'Connor HT, Sainsbury A, et al. Effect of resistance training on liver fat and visceral adiposity in adults with obesity: a randomized controlled trial. *Hepatol Res*. 2017;47(7):622-31.

Koo BK, Han KA, Ahn HJ, Jung JY, Kim HC, Min KW. The effects of total energy expenditure from all levels of physical activity vs. physical activity energy expenditure from moderate-to-vigorous activity on visceral fat and insulin sensitivity in obese Type 2 diabetic women. *Diabetic medicine : a journal of the British Diabetic Association*. Sep 2010;27(9):1088-92. doi:10.1111/j.1464-5491.2010.03045.x

Larson-Meyer et al., 2006, 2010; Redman et al., 2007, 2010

Larson-Meyer DE, Heilbronn LK, Redman LM, Newcomer BR, Frisard MI, Anton S, et al. Effect of calorie restriction with or without exercise on insulin sensitivity, beta-cell function, fat cell size, and ectopic lipid in overweight subjects. *Diabetes Care*. 2006;29(6):1337-44.

Larson-Meyer DE, Redman L, Heilbronn LK, Martin CK, Ravussin E. Caloric restriction with or without exercise: the fitness versus fatness debate. *Med Sci Sports Exerc*. 2010;42(1):152-9.

Redman LM, Heilbronn LK, Martin CK, Alfonso A, Smith SR, Ravussin E, et al. Effect of calorie restriction with or without exercise on body composition and fat distribution. *J Clin Endocrinol Metab*. 2007;92(3):865-72.

Redman LM, Veldhuis JD, Rood J, Smith SR, Williamson D, Ravussin E, et al. The effect of caloric restriction interventions on growth hormone secretion in nonobese men and women. *Aging Cell*. 2010;9(1):32-9.

Lee MG, Park KS, Kim DU, Choi SM, Kim HJ. Effects of high-intensity exercise training on body composition, abdominal fat loss, and cardiorespiratory fitness in middle-aged Korean females. *Appl Physiol Nutr Metab*. 2012;37(6):1019-27.

Lee YJ, Lee A, Yoo HJ, Kim M, Kim M, Jee SH, et al. Effect of weight loss on circulating fatty acid profiles in overweight subjects with high visceral fat area: a 12-week randomized controlled trial. *Nutr J*. 2018;17(1):28.

Lesser IA, Singer J, Hoogbruin A, Mackey DC, Katzmarzyk PT, Sohal P, et al. Effectiveness of exercise on visceral adipose tissue in older South Asian women. *Med Sci Sports Exerc*. 2016;48(7):1371-8.

Ng et al., 2007, 2009; Chan et al., 2008

Ng TWK, Watts GF, Barrett PHR, Rye KA, Chan DC. Effect of weight loss on LDL and HDL kinetics in the metabolic syndrome: associations with changes in plasma retinol-binding protein-4 and adiponectin levels. *Diabetes Care*. 2007;30(11):2945-50.

Ng TWK, Chan DC, Barrett PHR, Watts GF. Effect of weight loss on HDL-apoA-II kinetics in the metabolic syndrome. *Clin Sci*. 2009;118(1):79-85.

Chan DC, Watts GF, Ng TWK, Yamashita S, Barrett PHR. Effect of weight loss on markers of triglyceride-rich lipoprotein metabolism in the metabolic syndrome. *Eur J Clin Invest*. 2008;38(10):743-51.

Nordby et al., 2012; Bladbjerg et al., 2017

Nordby P, Auerbach PL, Rosenkilde M, Kristiansen L, Thomasen JR, Rygaard L, et al. Endurance training *per se* increases metabolic health in young, moderately overweight men. *Obesity*. 2012;20(11):2202-12.

Bladbjerg EM, Skov J, Nordby P, Stallknecht B. Endurance exercise *per se* reduces the cardiovascular risk marker t-PA antigen in healthy, younger, overweight men. *Thromb Res*. 2017;152:69-73.

Pugh et al., 2014; Cuthbertson et al., 2016

Pugh CJA, Sprung VS, Kemp GJ, Richardson P, Shojaee-Moradie F, Umpleby AM, et al. Exercise training reverses endothelial dysfunction in nonalcoholic fatty liver disease. *Am J Physiol Heart Circ Physiol*. 2014;307(9):H1298-306.

Cuthbertson DJ, Shojaee-Moradie F, Sprung VS, Jones H, Pugh CJA, Richardson P, et al. Dissociation between exercise-induced reduction in liver fat and changes in hepatic and peripheral glucose homoeostasis in obese patients with non-alcoholic fatty liver disease. *Clin Sci*. 2016;130(2):93-104.

Reichkendler MH, Auerbach P, Rosenkilde M, Christensen AN, Holm S, Petersen MB, et al. Exercise training favors increased insulin-stimulated glucose uptake in skeletal muscle in contrast to adipose tissue: a randomized study using FDG PET imaging. *Am J Physiol Endocrinol Metab*. 2013;305(4):E496-506.

Ross et al., 2000; Thong et al., 2000

Ross R, Dagnone D, Jones PJ, Smith H, Paddags A, Hudson R, et al. Reduction in obesity and related comorbid conditions after diet-induced weight loss or exercise-induced weight loss in men. A randomized, controlled trial. *Ann Intern Med*. 2000;133(2):92-103.

Thong FS, Hudson R, Ross R, Janssen I, Graham TE. Plasma leptin in moderately obese men: independent effects of weight loss and aerobic exercise. *Am J Physiol Endocrinol Metab*. 2000;279(2):E307-13.

Ross R, Janssen I, Dawson J, et al. Exercise-Induced Reduction in Obesity and Insulin Resistance in Women: a Randomized Controlled Trial. *Obesity research*. 2004;12(5):789-798. doi:https://doi.org/10.1038/oby.2004.95

Saremi A, Shavandi N, Parastesh M, Daneshmand H. Twelve-week aerobic training decreases chemerin level and improves cardiometabolic risk factors in overweight and obese men. *Asian J Sports Med*. 2010;1(3):151-8.

Schmitz KH, Hannan PJ, Stovitz SD, Bryan CJ, Warren M, Jensen MD. Strength training and adiposity in premenopausal women: strong, healthy, and empowered study. *Am J Clin Nutr*. 2007;86(3):566-72.

Schübel R, Nattenmüller J, Sookthai D, Nonnenmacher T, Graf ME, Riedl L, et al. Effects of intermittent and continuous calorie restriction on body weight and metabolism over 50 wk: a randomized controlled trial. *Am J Clin Nutr*. 2018;108(5):933-45.

Schutte S, Esser D, Siebelink E, et al. Diverging metabolic effects of two energy restricted diets differing in nutrient quality: a 12-week randomized controlled trial in subjects with abdominal obesity. *The American journal of clinical nutrition*. 2022:nqac025. doi:10.1093/ajcn/nqac025.

Shojaee-Moradie F, Baynes KCR, Pentecost C, Bell JD, Thomas EL, Jackson NC, et al. Exercise training reduces fatty acid availability and improves the insulin sensitivity of glucose metabolism. *Diabetologia*. 2007;50(2):404-13.

Slentz CA, Aiken LB, Houmard JA, Bales CW, Johnson JL, Tanner CJ, et al. Inactivity, exercise, and visceral fat. STRRIDE: a randomized, controlled study of exercise intensity and amount. *J Appl Physiol*. 2005;99(4):1613-8.

Trepanowski JF, Kroeger CM, Barnosky A, Klempel M, Bhutani S, Hoddy KK, et al. Effects of alternate-day fasting or daily calorie restriction on body composition, fat distribution, and circulating adipokines: secondary analysis of a randomized controlled trial. *Clin Nutr*. 2018;37(6 Pt A):1871-8.

Wu S, Park KS, McCormick JB. Effects of exercise training on fat loss and lean mass gain in Mexican-American and Korean premenopausal women. *Int J Endocrinol*. 2017;2017:5465869.

Zhang H, Tong T, Qiu W, Wang J, Nie J, He Y. Effect of high-intensity interval training protocol on abdominal fat reduction in overweight Chinese women: A randomized controlled trial. *Kinesiology*. 06/01 2015;47:57-66.

Zhang HJ, He J, Pan LL, Ma ZM, Han CK, Chen CS, et al. Effects of moderate and vigorous exercise on nonalcoholic fatty liver disease: a randomized clinical trial. *JAMA Intern Med*. 2016;176(8):1074-82.

Zhang H, Tong TK, Qiu W, Zhang X, Zhou S, Liu Y, et al. Comparable effects of high-intensity interval training and prolonged continuous exercise training on abdominal visceral fat reduction in obese young women. *J Diabetes Res*. 2017;2017:5071740.
